# Possible correlation of apical localization of MUC1 glycoprotein with luminal A-like status of breast cancer

**DOI:** 10.1038/s41598-023-32579-4

**Published:** 2023-03-31

**Authors:** Ryoko Semba, Yoshiya Horimoto, Madoka Sakata-Matsuzawa, Yumiko Ishizuka, Kaori Denda-Nagai, Haruhiko Fujihira, Miki Noji, Hiroko Onagi, Miyu Ichida, Hiroyoshi Miura, Junichiro Watanabe, Mitsue Saito, Tsuyoshi Saito, Atsushi Arakawa, Tatsuro Irimura

**Affiliations:** 1 Department of Breast Oncology, Juntendo University Faculty of Medicine, Tokyo, 113-0033 Japan; 2 Department of Human Pathology, Juntendo University Faculty of Medicine, Tokyo, Japan; 3 Intractable Disease Research Center, Juntendo University Graduate School of Medicine, Tokyo, Japan; 4 Division of Glycobiologics, Department of Breast Oncology, Juntendo University Faculty of Medicine, Tokyo, Japan; 5 Glycometabolic Biochemistry Laboratory, RIKEN Cluster for Pioneering Research, RIKEN, Wako, Japan; 6 Department of Surgery, Koshigaya Municipal Hospital, Saitama, Japan

**Keywords:** Breast cancer, Tumour biomarkers, Cancer, Surgical oncology

## Abstract

Adjuvant chemotherapy has played a major role in the treatment of hormone receptor-positive breast cancer for many years. To better determine which patient subsets need adjuvant chemotherapy, various gene expression analyses have been developed, but cost-effective tools to identify such patients remain elusive. In the present report, we retrospectively investigated immunohistochemical expression and subcellular localization of MUC1 in primary tumors and examined their relationship to tumor malignancy, chemotherapy effect and patient outcomes. We retrospectively examined three patient cohorts with hormone receptor-positive/human epidermal growth factor receptor 2-negative invasive breast cancer: 51 patients who underwent 21-gene expression analysis (multi-gene assay-cohort), 96 patients who received neoadjuvant chemotherapy (neoadjuvant chemotherapy-cohort), and 609 patients whose tumor tissue was used in tissue-microarrays (tissue-microarray-cohort). The immunohistochemical staining pattern of the anti-MUC1 monoclonal antibody, Ma695, was examined in cancer tissues, and subcellular localization was determined as apical, cytoplasmic or negative. In the multi-gene assay-cohort, tumors with apical patterns had the lowest recurrence scores, reflecting lower tumor malignancy, and were significantly lower than MUC1-negative tumors (*P* = 0.038). In the neoadjuvant chemotherapy-cohort, there was no correlation between MUC1 staining patterns and effects of chemotherapy. Finally, in the tissue-microarray-cohort, we found that patients with apical MUC1 staining patterns had significantly longer disease-free-survival and overall survival than other patterns (*P* = 0.020 and 0.039, respectively). Our data suggest that an apical MUC1 staining pattern indicates luminal A-likeness. Assessment of the subcellular localization of MUC1 glycoprotein may be useful for identifying patients who can avoid adjuvant chemotherapy.

## Introduction

In the treatment of hormone receptor (HR)-positive breast cancer, adjuvant chemotherapy has played a major part for many years. Risk assessment (i.e., the distinction between luminal A-like and B-like tumors) using factors, such as progesterone receptor (PgR) expression and the Ki67 labeling index, has been put into practical use^1^. In addition, to better determine the patient subsets needing adjuvant chemotherapy, various gene expression analyses, such as Oncotype Dx, have been developed^1,2^. However, such analyses are expensive and cost-effective tools to identify such patients are yet to be established. A glycoform of serum mucin 1 (MUC1), detected by a combination of monoclonal antibody (mAb) DF3 and mAb 115D8, has long been clinically used as CA15-3 to estimate the tumor burden of breast cancer^3^. Therefore, we decided to focus on the use of MUC1 as a clinicopathological indicator.

MUC1 is a transmembrane glycoprotein and is expressed in the apical membrane of glandular cells and luminal epithelial cells of the mammary gland, in addition to the esophagus, stomach, duodenum, pancreas, uterus, prostate and lung^4^. In normal tissue, MUC1 is believed to act as a physical barrier to protect the luminal surface of glandular cells from dryness, pH changes, contaminants, and microorganisms^5,6^. MUC1 consist of two subunits, a long *N*-terminal fragment (MUC1-N) and a short *C*-terminal fragment (MUC1-C)^7^. When the polarity of cells is lost in cancer, the polarity of the MUC1 display is also lost, and mucin expression increases, and the density and elongation of glycan changes^8^. In addition, the *N*-terminal portion of MUC1 is released into luminal spaces. MUC1 with such aberrant glycosylation is found in many cancer cells, such as in breast cancer, esophageal cancer, gastric cancer, ovarian cancer, and bladder cancer, and the released MUC1 is often found at high levels in the blood of patients with the above cancers.

Correlation of the cellular localization of MUC1 with breast cancer prognosis was first reported by Ceriani and co-workers using mAb BrE-3^9^. This antibody was found to recognize the amino acid residue, TRP, within the tandem repeat of MUC1, and the extension of glycan attached to the threonine residue seems to prevent its binding^10^. Further evaluation of the significance of the cytoplasmic localization of MUC1 detected by mAbs specific for the peptide portion of MUC1 tandem repeats, further confirmed the strong correlation^11^. However, the clinicopathological use of this subcellular localization of MUC1 in breast cancer is yet to be evaluated, particularly under the advanced framework of current therapeutic options. In the present report, we retrospectively investigated the expression and subcellular localization of MUC1 by using mAb Ma695, which recognizes MUC1 with a sialyl-T glycan attached to the threonine residue of the GVTS sequence within the tandem repeats^12^. The binding of this antibody was immunohistochemically determined in primary tumors and its relationship to tumor malignancy, effects of chemotherapy and patient outcomes was examined.

## Material and methods

### Patients

We examined three patient cohorts with HR-positive/human epidermal growth factor receptor (HER) 2-negative invasive breast cancer in the current study. The first cohort was chosen to investigate the relationship between anti-MUC1 mAb staining patterns and the degree of tumor malignancy. Fifty-one patients who underwent 21-gene expression analysis for breast cancer diagnosis (Oncotype DX, Genomic Health, Redwood City, CA) after curative surgery during the period from 2017 to 2021 were enrolled (multi-gene assay [MGA]-cohort). This MGA was developed for HR-positive and HER2-negative breast cancer patients to identify those who are likely to obtain benefit from adjuvant chemotherapy^2^. Recurrence score (RS) is used as the indicator for this assay, ranging from 0 to 100, where higher scores correspond to a worse prognosis and a likely benefit from adjuvant chemotherapy. The second cohort was used to investigate the relationship between anti-MUC1 mAb staining patterns and the outcomes of chemotherapy. Ninety-six patients who received neoadjuvant chemotherapy (NAC) and underwent curative surgery during the period from 2006 to 2008 were enrolled and retrospectively examined (NAC-cohort). The third cohort was used to examine the relationship between anti-MUC1 mAb staining patterns and the clinical outcomes of patients. Tissue-microarrays (TMAs) from tumors of 609 patients who underwent curative surgery from 2014 through 2019 were used (TMA-cohort). TMAs were constructed using 2.0 mm cores sampled from one representative area of each formalin-fixed paraffin-embedded tissue block from primary tumors, as described previously^13^. This cohort included four patients who had estrogen receptor-negative/PgR-positive tumors. The clinicopathological backgrounds of these three cohorts are shown in Supplementary Table 1.

This study was performed with approval from the Ethics Committee of Juntendo University Hospital (H19-0289). Patients could see the research plan on the hospital website and were offered the choice to opt out of the study at any time. All data were anonymized before use.

### Pathological assessment

Pathological examinations were carried out at Juntendo University Hospital by two experienced pathologists. Tumor grade was judged based on the modified Bloom-Richardson histological grading system. For patients who received NAC, a pathological complete response (pCR) was defined as the disappearance of invasive nests both in the dissected primary breast tumor and lymph nodes. Estrogen receptor and PgR statuses were assessed semi-quantitatively with immunohistochemistry and reported as positive when > 1% of cancer cell nuclei were stained. HER2 was judged as positive when more than 10% of tumor cells showed strong staining of the entire cell membrane, or *HER2/neu* gene amplification was observed by fluorescence in situ hybridization. We excluded HER2-positive cases from the current study. The Ki67 labeling index was semi-quantitatively evaluated by the percentage of cells positive for nuclear Ki67 within a selected hotspot microscopically under high magnification.

**Figure 1:**
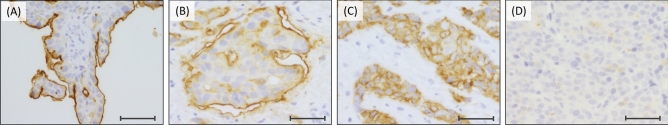
Representative images of MUC1 staining. Representative images of MUC1 staining are shown. (**A**) Apical, (**B**) apical + cytoplasmic, (**C**) cytoplasmic, (**D**) negative. The horizontal bar indicates 100 µm.

Binding profiles with anti-MUC1 mAb Ma695 (Leica Biosystems, Tokyo, Japan) were immunohistochemically examined. Biopsy specimens prior to systemic treatments were used for the NAC-cohort. Surgical specimens were used for the MGA- and TMA-cohorts. Specimens were cut at 3 μm thickness from formalin-fixed paraffin-embedded samples for immunohistochemistry. The staining patterns in cancer tissues were assessed and classified as apical (Ap), apical + cytoplasmic (Ap + Cy), cytoplasmic (Cy), or negative, according to the methods employed in previous reports^14,15^. Representative images are shown in Fig. 1. The classifications, Ap and Ap + Cy, were made when more than 10% of cancer cells examined had these patterns.

### Statistical assessment

Statistical analyses were performed using JMP 11.2.1 statistical software (SAS Institute Inc., Cary, NC). For comparisons of mean values among multiple groups and two groups, analysis of variance and two-sided *t* tests were employed, respectively. As a test of independence, the Pearson’s Chi-squared test was used. A logistic regression model was constructed to identify factors characteristic to pCR cases. For evaluation of the independent prognostic effects of the staining profiles, the Cox proportional hazard model was applied with a 95% confidence interval. In the NAC-cohort, the full-model analysis selected variables according to their clinical significance. Sample size, age, pathological size of the invasive disease, lymph node metastasis, Ki67 labeling index, PgR status, and distribution of anti-MUC1 mAb staining were chosen. Kaplan–Meier curves were drawn, and log-rank tests were applied to compare the survival curves of the two populations. A *P*-value < 0.050 was considered statistically significant.

### Ethics approval

All procedures performed in studies involving human participants were in accordance with the ethical standards of the Ethics Committee of Juntendo University Hospital (H19-0289) and with the 1964 Helsinki declaration and its later amendments or comparable ethical standards. The requirement for informed consent was waived by the Ethics Committee of Juntendo University Hospital.

### Consent to participate

An opt-out approach was used with the disclosure on our website.

## Results

### MUC1 staining patterns in the MGA-cohort

**Figure 2:**
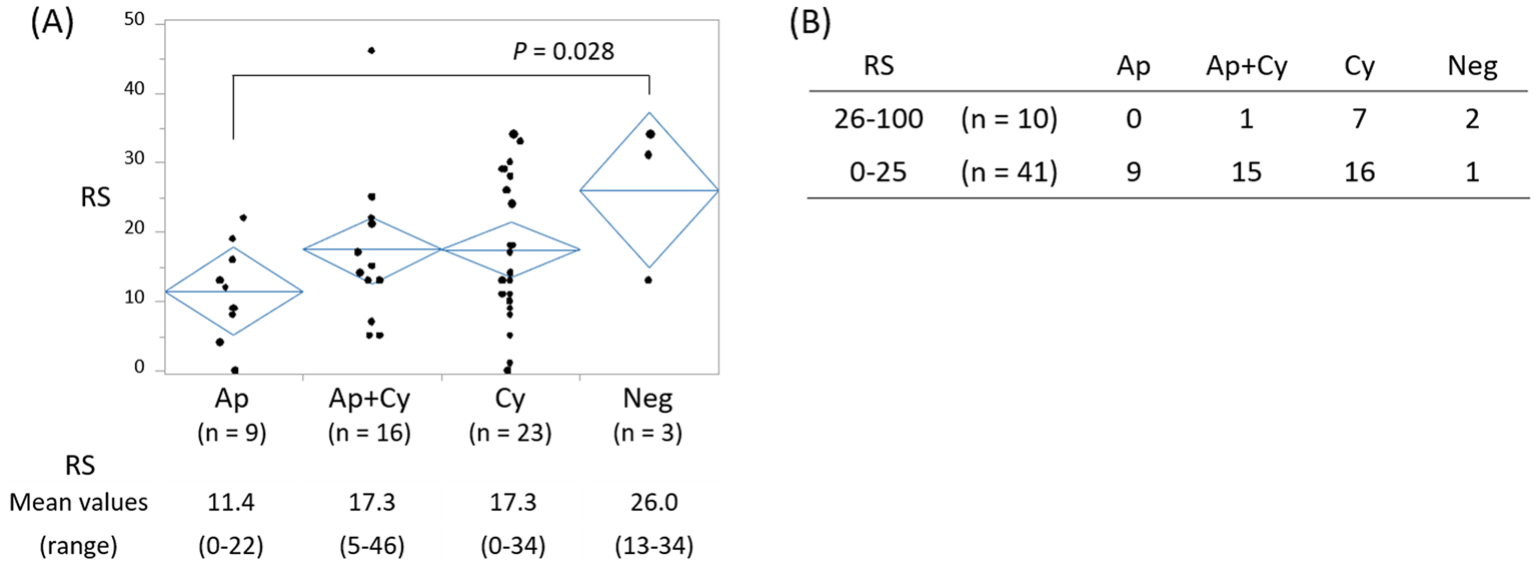
MUC1 staining patterns and recurrence score. (**A**) Recurrence score (RS) according to the MUC1 staining pattern. Horizontal blue lines indicate the mean values of each group. (**B**) RS distribution according to MUC1 staining when patients were divided into two groups, high and low RS. *Ap* apical, *Ap* + *Cy* apical + cytoplasmic, *Cy* cytoplasmic, *Neg* negative.

In the MGA-cohort (n = 51), RS was available for each patient and the mean RS was 16.8 (range, 0–46). Staining patterns with anti-MUC1 mAb Ma695 in the 51 tumors differed among patients and the proportions of Ap, Ap + Cy Cy and negative were 18%, 31%, 45% and 6%, respectively. The relationship between RS and MUC1 staining patterns is shown in Fig. 2A. Tumors with the Ap pattern had the lowest RS (11.4) and RS increased as apical staining was lost, as MUC1-negative tumors had the highest RS (26.0). The RS of Ap-pattern tumors was significantly lower than that of MUC1-negative tumors (*P* = 0.028), while there was no significant difference in RS when all four groups were compared (*P* = 0.144).

RSs were then categorized into two groups, high (26–100) and low (0–25), based on several large clinical trials^16,17^. The percentage of tumors with a high RS and low RS was 20% (10 patients) and 80% (41 patients), respectively. Under this categorization, a significant correlation between RS and MUC1 staining patterns was also observed (Fig. 2B, P = 0.019) and all tumors with the Ap pattern were categorized into the low RS group.

Additionally, this cohort included two patients with invasive micropapillary carcinoma (IMPC) and the MUC1 staining patterns were Ap and Ap + Cy in these two patients.

### MUC1 staining patterns in the NAC-cohort

Staining patterns of MUC1 in the 96 tumors in this cohort were Ap in 7%, Ap + Cy in 41%, Cy in 48% and negative in 4% of tumors. Table 1 shows the relationship between the effects of chemotherapy and the anti-MUC1 mAb staining profiles, together with other clinicopathological features. Among the variables examined, only the Ki67 labeling index was an independent factor relating to pCR, as patients with pCR had significantly higher Ki67 labeling indexes in biopsy specimens (*P* = 0.033). There was no correlation between MUC1 staining patterns and the effects of chemotherapy on the whole, but there was no case showing pCR in the Ap group. Moreover, Supplementary Table 2 shows the relationship between MUC1 staining patterns and clinicopathological factors in the NAC-cohort. Tumors with Ap staining patterns were significantly smaller than the others (*P* < 0.001), while there was no difference in other factors, including the Ki67 labeling index.

**Table 1:** Relationship between the effect of chemotherapy and clinicopathological features in the neoadjuvant chemotherapy cohort (n = 96).

Variables	pCR	Non-pCR	Univariate			Multivariate		
	n = 5	n = 91	OR	95% CI	*P*-value	OR	95% CI	*P*-value
Age, mean, years	55.2	51.5	5.2	0.1–740.7	0.479	2.3	0.02–2.4E+02	0.723
Histology								
NST	5	87	6.3E+05	1.2E+90–∞	0.508	4.0E+07	0.02–∞	0.999
Others	0	4						
Tumour grade								
High	1	9	2.3	0.1–17.4	0.519	1.5	0.1–20.9	0.748
Intermediate/low	4	81						
Ki67 L.I., mean, %	43	24	42.5	1.4–1.3E+3	0.039	71.4	1.4–3.6E+03	0.033
PgR								
Positive	2	67	0.2	0.03–1.5	0.116	0.2	0.02–2.0	0.176
Negative	3	23						
MUC1 staining								
Ap	0	7	1.7E−08^a^	0.0–6.4	0.378	1.2E−07^a^	0.0–9.0	0.999
Ap + Cy	1	38						
Cy	3	43						
Negative	1	3						

All biomarkers were assessed based on biopsy specimens.

^a^Compared in two groups between Ap and other patterns.

*pCR* pathological complete response, *NST* no special type, *L.I.* labelling index, *PgR* progesterone receptor, *OR* odds ratio, *CI* confidence interval, *Ap* apical, *Cy* cytoplasmic.

As for patient outcomes in the NAC-cohort, 27 of the 96 patients (28%) developed distant metastasis and 17 patients died due to breast cancer during the median 118-month observation period (range, 9–191 months). Supplementary Table 3 shows the relationship between disease-free-survival (DFS) and overall survival (OS), and the clinicopathological features. Age, pathological invasive size of remnant disease, and PgR status were related with DFS (*P* = 0.031, 0.016 and 0.031, respectively), as young age, patients with large and PgR-negative tumors had significantly shorter DFS. The pathological invasive size of remnant disease and PgR status were also associated with OS (*P* = 0.021 and 0.019, respectively).

### MUC1 staining patterns in the TMA-cohort

Staining patterns of MUC1 in the 609 tumors from the TMA-cohort were Ap in 10%, Ap + Cy in 34%, Cy in 50% and negative in 5% of tumors. There were seven patients with IMPC tumors included in this cohort and MUC1 staining patterns were Ap in five tumors and Ap + Cy in two tumors. The relationship between MUC1 staining patterns and clinicopathological factors in the TMA-cohort are shown in Supplementary Table 4. Patients with Ap-pattern tumors were younger and more frequently had special histological types of tumors, compared with other staining patterns (*P* = 0.027 and 0.002, respectively). Among these special histological types, mucinous carcinoma and IMPC frequently had Ap MUC1 staining patterns (35% and 71%, respectively), while no invasive lobular carcinomas had Ap patterns.

**Figure 3:**
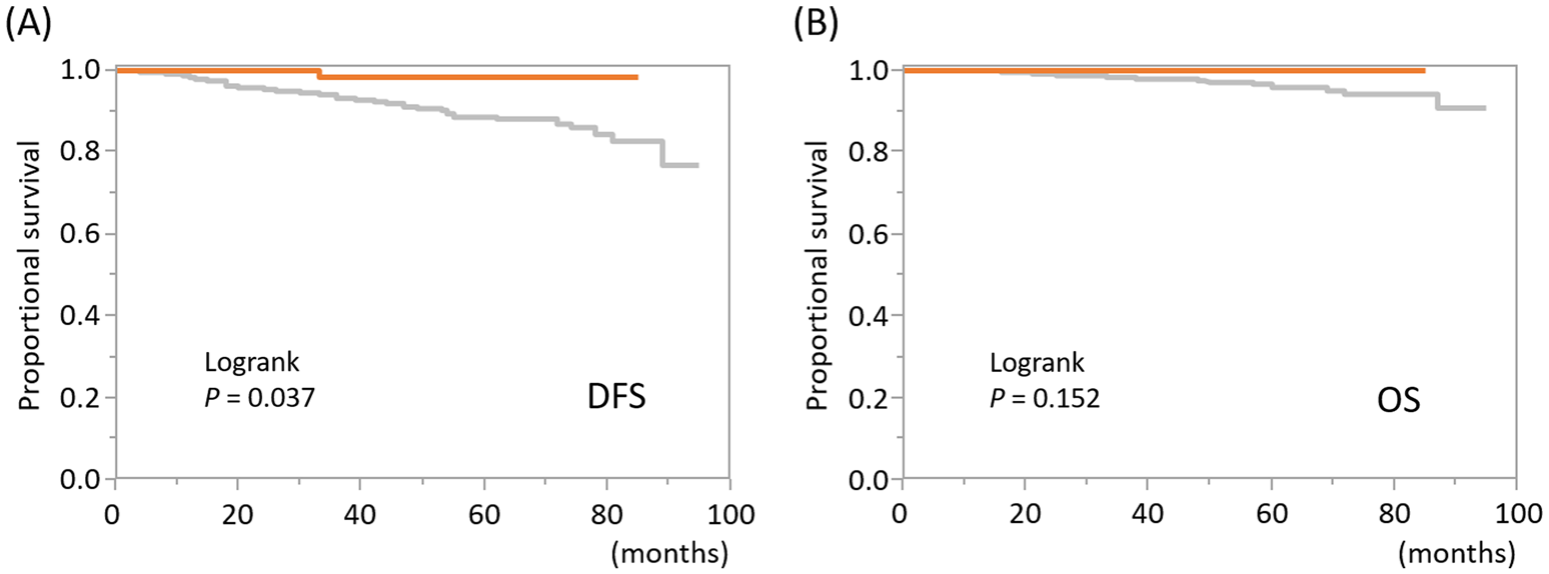
Kaplan–Meier curves of patient outcomes according to MUC1 staining pattern. Kaplan–Meier curves of (**A**) disease-free survival (DFS) and (**B**) overall survival (OS) according to MUC1 staining pattern are shown. Orange curves indicate patients with tumors with an apical MUC1 staining pattern, while grey curves indicate patients with tumors with other staining patterns.

During the median 53-month observation period (range, 1–95 months), 55 of the 609 patients (9%) developed distant metastasis and 18 patients (3%) died due to breast cancer. Table 2 shows the relationship between DFS and OS, and clinicopathological features according to results of the Cox proportional hazard model. Tumor size, PgR status, and MUC1 staining were independent factors relating to DFS. Patients with large tumors had shorter DFS (*P* = 0.025), while those with PgR-positive tumors and tumors with Ap MUC1 staining patterns had significantly longer DFS (*P* = 0.013, and 0.020, respectively). Patients with either large, high-grade or PgR-negative tumors had shorter OS (*P* = 0.008, 0.006 and 0.034, respectively). Meanwhile, patients with Ap-pattern tumors had significantly longer OS (*P* = 0.039). Finally, we drew Kaplan–Meier curves according to the MUC1 staining pattern (Fig. 3**)**. A log-rank test revealed that Ap-pattern tumors had significantly longer DFS (*P* = 0.037) compared with other patterns. Meanwhile, there was no difference in OS (*P* = 0.152), which was different to the results of the Cox proportional hazard model. Kaplan–Meier curves of patient outcomes compared among the four patterns of MUC1 staining are shown in Supplementary Fig. 1.

**Table 2:** Relationship between patient outcomes and clinicopathological features in the tissue microarray-cohort.

Variables	Univariate			Multivariate		
	HR	95% CI	*P*-value	HR	95% CI	*P*-value
Disease-free survival						
Age	0.53	0.13–2.13	0.379	0.49	0.10–2.51	0.396
Tumor size	19.22	4.41–66.67	< 0.001	8.42	1.35–38.77	0.025
Lymph node metastasis						
Yes vs. no	2.77	1.58–5.03	< 0.001	1.58	0.83–3.07	0.165
Histology						
NST vs. others	0.98	0.50–2.14	0.950	0.99	0.38–3.40	0.989
Tumor grade						
High vs. intermediate/low	3.12	1.66–5.56	< 0.001	1.89	0.94–3.66	0.074
Ki67 L.I	6.91	2.36–19.76	< 0.001	2.84	0.79–9.77	0.109
ER						
Positive vs. negative	6.64E+07	0.16–0.16	0.435	4.22E+09	0.36-	0.222
PgR						
Positive vs. negative	0.31	0.16–0.63	0.002	0.34	0.16–0.78	0.013
Administration of chemotherapy						
Yes vs. no	3.71	2.15–6.59	< 0.001	1.98	0.98–4.13	0.058
MUC1 staining						
Ap vs. others	0.16	0.01–0.73	0.012	0.17	0.01–0.80	0.020
Overall survival						
Age	0.27	0.02–3.16	0.303	0.16	0.01–2.75	0.206
Tumor size	9.32E+01	11.61–526.30	< 0.001	51.50	3.18–560.57	0.008
Lymph node metastasis						
Yes vs. no	4.79	1.70–17.03	0.003	2.30	0.66–9.60	0.196
Histology						
NST vs. others	0.38	0.15–1.10	0.072	0.32	0.08–1.60	0.151
Tumor grade						
High vs. intermediate/low	7.85	2.88–21.39	< 0.001	5.78	1.71–19.43	0.006
Ki67 L.I	10.16	1.60–62.92	0.015	3.38	0.33–29.77	0.297
ER						
Positive vs. negative	2.44E+07	0.03–0.03	0.703	1.70E + 12	8.9E+10–2.8E+13	0.624
PgR						
Positive vs. negative	0.20	0.08–0.63	0.008	0.21	0.06–0.88	0.034
Administration of chemotherapy						
Yes vs. no	6.43	2.30–22.68	< 0.001	1.13	0.30–4.89	0.862
MUC1 staining						
Ap vs. others	1.72E−09	0.99–0.99	0.049	2.66E−10	0–0.89	0.039

*HR* hazard ratio, *CI* confidence interval, *NST* no special type, *L.I.* labelling index, *ER* estrogen receptor, *PgR* progesterone receptor, *Ap* apical.

## Discussion

MUC1 is the major constituent of milk fat globule membranes secreted from normal mammary ductal epithelial cells^6^. Glycoforms of MUC1 (MUC1 with different glycans) present in sera have long been used as serum markers for carcinomas and interstitial pneumonitis^18,19^. However, debate surrounds its use as a tissue marker for breast malignancy, with one study reporting that genes induced by MUC1 worsen the outcome of breast cancer patients^20^, while others show that MUC1 expression correlates to a better prognosis^15,21–23^.

Cellular localization patterns of MUC1 in breast cancer were previously reported to differ depending on the type of tumor and the use of mAbs with different specificities^9,11,14,15,24^. MUC1 localizes in the cytoplasm, cell surface membranes and intracellular membranes. These localization patterns may differ depending on the breast cancer subtype^14,24^. The relationship between the localization of MUC1 and patient prognosis was reported as early as 1991^9,11^, but MUC1 distribution patterns and therapeutic outcomes have not been evaluated using intrinsic subtyping and precision medicine with genetic profiling.

In our present study of the MGA-cohort, we found that the RS obtained from gene expression profiles corresponds to the subcellular localization of MUC1 revealed by the binding of mAb Ma695. Tumors having Ap patterns in MUC1 staining had the lowest RS. To the best of our knowledge, this is the first report showing such a relationship. The 21-gene multi-gene expression profiling (Oncotype Dx) used in the present study consists of 16 important genes involved in cancer biology, such as proliferation, infiltration and the estrogen signaling pathway, and has been developed to identify patients who may benefit from adjuvant chemotherapy in HR-positive cancer^2,16,17^. It is also used as a tool to predict patient prognosis^16,17,25–27^. Therefore, our present results suggest that cellular localization of MUC1 with a unique glycosylation is potentially useful as a prognostic indicator of luminal-type breast cancer, as comprehensively reviewed by Pinho and Reis^28^.

In our studies of the NAC-cohort, there was no pCR in Ap-pattern tumors. Therefore, chemotherapy was not effective for patients with Ap-pattern tumors. This cohort included patients from an earlier period when NAC was still given to many patients with luminal-type breast cancers. Therefore, the results are valuable because such a population cannot be studied under the current chemotherapy standards.

Patients with tumors having the Ap pattern in MUC1 staining had the best outcomes in our TMA-cohort of 609 patients. Our data showed that comparing patients with Ap patterns to those with other staining patterns in MUC1 staining seemed to be useful for prognosis prediction. Previously, Ceriani and colleagues reported that patients with low cytoplasmic and high membrane intensity, when stained with mAb BrE-3, specific for the TRP sequence in the tandem repeat of MUC1, were associated with a good prognosis^9^. Rahn and co-workers evaluated tumors in 71 breast cancer patients by immunohistochemical staining with mAb B27.29, specific for an epitope PDTRPAP in the tandem repeat of MUC1, and found that tumors with poor staining in the cytoplasm had a good prognosis^11^. In these early studies, HER2 or HR status was unknown in most cases. No study has ever clarified a prognostic association of MUC1 cellular localization patterns using mAb Ma695, a mAb specific for MUC1 with sialyl-T residues, for luminal HER2-negative breast cancer on the scale of our study. It should be emphasized that the results of the present study are directly applicable under the current framework of breast cancer treatment.

The mechanism of how glycosylated MUC1 exhibits cytoplasmic localization in tumor cells of some cases of luminal-type breast cancer is presently unknown. Kinlough and colleagues showed that apical localization of MUC1 was dependent on the extension of *O-*glycans, and blockade of this extension by sialylation of position 6 of *N*-acetylgalactosamine (GalNAc) induced cytoplasmic localization of MUC1^29^. The importance of such a mechanism is worth further investigation, though MUC1 with this epitope, sialyl-Tn, is not likely to be recognized by mAb Ma695^12^.

The mechanistic correlation of cytoplasmic localization with elevated malignant behavior of luminal-type breast cancer cells is another important question. The function of MUC1 in cancer might change according to its intracellular localization. For example, when the localization of MUC1 shifts, it decreases cell-to-cell adhesion via binding to β-catenin^30^. As a result, cancer migration and invasion may be promoted^31,32^. Moreover, by boosting the interaction with molecules normally expressed on the basement membrane, such as epidermal growth factor receptor, various intracellular signaling pathways could be activated^33^.

MUC1 expression in the apical membrane is likely to represent a luminal A-like phenotype (low grade cancer resulting in the most favorable prognosis). Our data showing a low RS and better patient survival in tumors with apical MUC1 localization support this possibility. Therefore, assessment of MUC1 subcellular localization may be useful in selecting patients who can avoid adjuvant chemotherapy in clinical practice. Luminal formation is one factor in histological grading, but this structure does not always show an Ap staining pattern with anti-MUC1 mAb Ma695. Thus, we believe that evaluation of MUC1 localization by immunohistochemical staining has the potential to be a simple and cost-effective option to estimate the aggressiveness of luminal-type breast cancer.

While invasive lobular carcinomas showed no Ap staining pattern, all IMPC tumors showed apical staining (Ap or Ap + Cy) in the current study. Considering this histological type is known to have relatively poor patient outcomes^34^, the MUC1 staining pattern might have to be interpreted separately for such tumors with special histological features in future studies.

Many tumors indeed had a mixed pattern of Ap and Cy. However, we could not manage to explore the intra-tumor heterogeneity of the MUC1 staining pattern. Intra-tumor heterogeneity is of major importance. It is thought to be created by the cancer cells themselves at the genetic and epigenetic levels during various stages, from carcinogenesis to the development of metastatic disease^35,36^. Through inter-clonal cooperation, tumor heterogeneity might functionally contribute to adaptation of the tumor microenvironment, tumor progression and resistance to therapy. We believe it is possible that heterogeneity in MUC1 reactivity may also have biological or clinical significance, and it should be clarified by further research.

One of the other limitations of our present study is the specificity of the mAb Ma695^12^. Combinations of mAbs with different specificity may increase the accuracy of the prognostic prediction. It has been reported that MUC1-C contributes to resistance to tamoxifen^37^, however, we were not able to directly verify the relationship between MUC1 localization and the effect of endocrine therapy in the present study. Another study limitation was the small sample size. Especially in the MGA-cohort, the number of RS-high cases was small. Further studies with a larger sample size may be needed to confirm the association of the MUC1 staining pattern with tumor malignancy. In addition, the observation period of our TMA-cohort was relatively short, and the relationship with late recurrence could not be investigated. Whether or not Ap-pattern tumors tend to develop late recurrence is of great interest and this issue is to be addressed in the future. Finally, there is the concern of the assessment of MUC1 staining. The three cohorts we employed in this study differed in terms of the area to be evaluated. Considering the possible heterogeneity of MUC1 staining, the interpretation of results may require caution, especially for the NAC- and TMA-cohorts where we were forced to evaluate small samples.

In conclusion, our data suggest that intracellular localization of MUC1 could be a prognostic factor in luminal HER2-negative breast cancer. The Ap pattern in MUC1 staining may indicate luminal A-likeness, and assessment of the subcellular localization of MUC1 may be important from the perspective of screening patients who can avoid adjuvant chemotherapy in clinical practice.

## Supplementary Information

Supplementary Figure 1.

Supplementary Table 1.

Supplementary Table 2.

Supplementary Table 3.

Supplementary Table 4.

## Data Availability

All data generated or analyzed during this study are included in this published article.
