# Supplementary material for: Possible correlation of apical localization of MUC1 glycoprotein with luminal A-like status of breast cancer
Source: Sci Rep. 2023 Mar 31;13:5281. doi: 10.1038/s41598-023-32579-4 (PMC10066179; doi:10.1038/s41598-023-32579-4)
Supplement: Supplementary file 1 — Supplementary Figure 1. [file 41598_2023_32579_MOESM1_ESM.pdf]

Supplementary Figure 1. Kaplan-Meier curves according to the four patterns of MUC1 staining

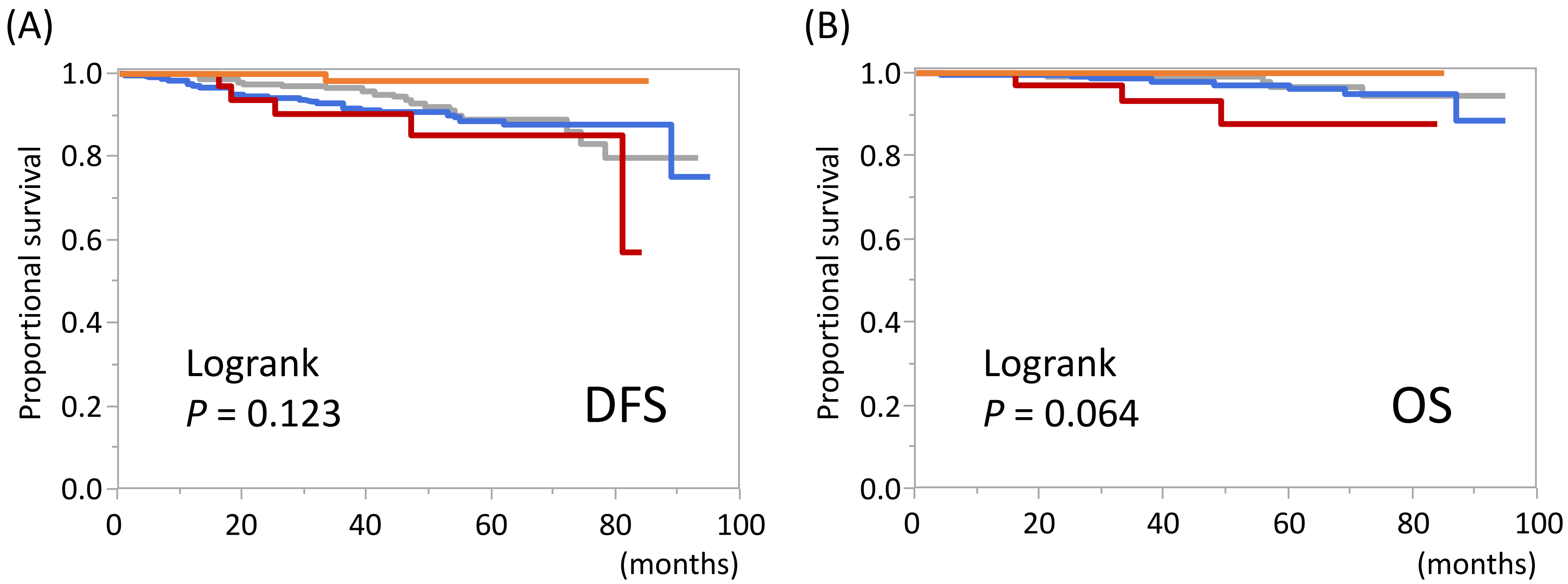

Kaplan-Meier curves of (A) disease-free survival (DFS) and (B) overall survival (OS) according to the four patterns of MUC1 staining are shown. Orange, grey, blue and red curves indicate patients with tumors with apical, apical+cytoplasmic, cytoplasmic and negative staining patterns, respectively.
