# Supplementary material for: Possible correlation of apical localization of MUC1 glycoprotein with luminal A-like status of breast cancer
Source: Sci Rep. 2023 Mar 31;13:5281. doi: 10.1038/s41598-023-32579-4 (PMC10066179; doi:10.1038/s41598-023-32579-4)
Supplement: Supplementary file 2 — Supplementary Table 1. [file 41598_2023_32579_MOESM2_ESM.pdf]

Supplementary Table 1 Clinicopathological features of the three cohorts examined

(**A**) Multi-gene assay (MGA)-cohort

Variable			MGA-cohort (n = 51)	
Age, mean [range], y			52.0	[31-76]
Histology	NST		45	(88)
	Others -		6	(12)
		ILC	3	
		IMPC	2	
		ICC	1	
pT, mean [range], mm			25.0	[8-75]
pN	Positive		25	(49)
	Negative		26	(51)
pStage	I		17	(33)
	II		31	(61)
	III		3	(6)
Tumour grade	High		9	(18)
	Intermediate/low		42	(82)
Ki67 L.I., mean [range],%			39.2	[5-80]
ER	Positive		51	(100)
	Negative		0	(0)
PgR	Positive		51	(100)
	Negative		0	(0)

Values are n (%) unless otherwise indicated.
NST no special type, *ILC* invasive lobular carcinoma, *IMPC* invasive micropapillary carcinoma, ICC invasive cribriform carcinoma, L.I. labelling index, ER estrogen receptor, PgR progesterone receptor

(**B**) Neoadjuvant chemotherapy (NAC)-cohort

Variable		NAC-cohort (n = 96)	
Age, mean [range], y		51.8	[22-78]
Histology	NST	92	(96)
	Others -	4	(4)
	ILC	4	
ypT, mean [range], mm		28.7	[0-110]
ypN	Positive	61	(64)
	Negative	35	(36)
ypStage	0	5	(5)
	I	17	(18)
	II	50	(52)
	III	24	(25)
Tumour grade	High	10	(10)
	Intermediate/low	85	(89)
	Unknown	1	(1)
Ki67 L.I., mean [range],%		24.8	[2-90]
ER	Positive	96	(100)
	Negative	0	(0)
PgR	Positive	69	(72)
	Negative	26	(27)
	Unknown	1	(1)
NAC regimen	A+T	90	(94)
	A alone	5	(5)
	T alone	1	(1)

Values are n (%) unless otherwise indicated.

NST no special type, *ILC invasive lobular carcinoma, L.I.* labelling index,
ER estrogen receptor, PgR progesterone receptor, A anthracycline, T taxane

(**C**) Tissue microarray (TMA)-cohort

Variable			TMA-cohort (n = 609)	
Age, mean [range], y			56.3	[28-93]
Histology	NST		507	(83.2)
	Others -		102	(16.8)
		ILC	52	
		Mucinous	31	
		IMPC	7	
		ICC	4	
		Tubular	4	
		Apocrine	2	
		Medullary	1	
		Squamous	1	
pT, mean [range], mm			27.6	[2-139]
pN	Positive		232	(38)
	Negative		333	(55)
	Not evaluated		44	(7)
pStage	I		186	(31)
	II		355	(58)
	III		68	(11)
Tumour grade	High		72	(12)
	Intermediate/low		494	(81)
	Not evaluated		43	(7)
Ki67 L.I., mean [range],%			32.8	[0-100]
ER	Positive		605	(99)
	Negative		4	(1)
PgR	Positive		556	(91)
	Negative		53	(9)
Adjuvant chemotherapy	Yes		205	(34)
	No		404	(66)

NST no special type, *ILC* invasive lobular carcinoma, *IMPC* invasive micropapillary carcinoma, ICC invasive cribriform carcinoma, L.I. labelling index, ER estrogen receptor, PgR progesterone receptor
