# Supplementary material for: Possible correlation of apical localization of MUC1 glycoprotein with luminal A-like status of breast cancer
Source: Sci Rep. 2023 Mar 31;13:5281. doi: 10.1038/s41598-023-32579-4 (PMC10066179; doi:10.1038/s41598-023-32579-4)
Supplement: Supplementary file 3 — Supplementary Table 2. [file 41598_2023_32579_MOESM3_ESM.pdf]

Supplementary Table 2 Relationship between MUC1 staining pattern and clinicopathological features in the NAC-cohort (n = 98)

Variables		MUC1 staining		*P* -value
		Ap	Others	
Age, mean, y		50.6	51.8	0.723
Histology	NST	7	85	0.567
	Others	0	4*	
ypT, mean, mm		15.3	29.8	< 0.001
ypN	Positive	5	56	0.653
	Negative	2	33	
Tumour grade	High	2	8	0.106
	Intermediate/low	5	80	
Ki67 L.I., mean,%		23.6	24.8	0.841
PgR	Positive	5	64	0.941
	Negative	2	24	

MUC1 mucin 1, *NAC* neoadjuvant chemotherapy, *Ap* apical, *NST* no special type, *L.I.* labelling index, *PgR* progesterone receptor

*All were invasive lobular carcinoma
