# Supplementary material for: Possible correlation of apical localization of MUC1 glycoprotein with luminal A-like status of breast cancer
Source: Sci Rep. 2023 Mar 31;13:5281. doi: 10.1038/s41598-023-32579-4 (PMC10066179; doi:10.1038/s41598-023-32579-4)
Supplement: Supplementary file 4 — Supplementary Table 3. [file 41598_2023_32579_MOESM4_ESM.pdf]

Supplementary Table 3 Relationships between patient outcomes and clinicopathological features in the NAC-cohort

Disease-free survival

Variables		Univariate HR	Univariate 95% CI	Univariate *P*-value	Multivariate HR	Multivariate 95% CI	Multivariate *P*-value
Age		0.14	0.02-1.04	0.055	0.09	0.01-0.80	0.031
Chemo-effect	pCR vs. non-pCR	1.88E-09	1.29-1.29	0.083			
ypT (mm)		20.32	2.79-113.88	0.004	12.93	1.68-83.97	0.016
ypN	Positive vs. negative	2.04	0.88-5.56	0.102	1.59	0.63-4.56	0.338
Histology	NST vs. others	5.46E+08	0.87-0.87	0.066			
Tumour grade	High vs. intermediate/low	0.94	0.22-2.72	0.919			
Ki67 L.I.		0.80	0.10-4.58	0.819	1.34	0.15-9.20	0.784
PgR	Positive vs. negative	0.61	0.28-1.38	0.223	0.38	0.17-0.91	0.031
MUC1 staining	Ap vs. others	0.39	0.02-1.88	0.290	0.45	0.02-2.23	0.386

Overall survival (breast cancer-related)

Variables		Univariate HR	Univariate 95% CI	Univariate *P*-value	Multivariate HR	Multivariate 95% CI	Multivariate *P*-value
Age		0.11	0.01-1.51	0.100	0.07	0.004-1.23	0.069
Chemo-effect	pCR vs. non-pCR	5.10E-09	2.17-2.17	0.176			
ypT (mm)		20.65	2.60-127.57	0.006	14.43	1.52-124.18	0.021
ypN	Positive vs. negative	2.55	0.83-11.06	0.107	2.13	0.55-11.39	0.286
Histology	NST vs. others	5.46E+08	0.54-0.54	0.143			
Tumour grade	High vs. intermediate/low	0.63	0.20-2.75	0.489			
Ki67 L.I.		1.66	0.16-11.10	0.644	5.43	0.37-64.84	0.206
PgR	Positive vs. negative	0.50	0.19-1.38	0.174	0.27	0.09-0.80	0.019
MUC1 staining	Ap vs. others	1.64E-09	1.09-1.09	0.060	1.53E-10	0.00-1.60	0.113

All biomarkers were assessed based on biopsy specimens

NAC neoadjuvant chemotherapy, *pCR* pathological complete response, *NST* no special type, *L.I*. labelling index, *HR* harazd ratio, *CI* confidence interval, *PgR* progesterone receptor, *MUC1* mucin 1, *Ap* apical
