# Supplementary material for: Possible correlation of apical localization of MUC1 glycoprotein with luminal A-like status of breast cancer
Source: Sci Rep. 2023 Mar 31;13:5281. doi: 10.1038/s41598-023-32579-4 (PMC10066179; doi:10.1038/s41598-023-32579-4)
Supplement: Supplementary file 5 — Supplementary Table 4. [file 41598_2023_32579_MOESM5_ESM.pdf]

Supplementary Table 4 Relationship between MUC1 staining pattern and clinicopathological features in the TMA-cohort (n = 609)

Variables			MUC1 staining		*P* -value
			Ap	Others	
Age, mean, y			52.7	56.7	0.027
Histology	NST		43	464	0.002
	Others -		19	83	
		ILC	0	52	
		Mucinous	11	20	
		IMPC	5	2	
		ICC	2	2	
		Tubular	1	3	
		Apocrine	0	2	
		Medullary	0	1	
		Squamous	0	1	
pT, mean, mm			27.2	27.6	0.873
pN	Positive		19	213	0.118
	Negative		41	292	
Tumour grade	High		10	62	0.275
	Intermediate/low		48	446	
Ki67 L.I., mean,%			34.3	32.6	0.604
ER	Positive		62	543	0.499
	Negative		0	4	
PgR	Positive		56	500	0.774
	Negative		6	47	
Adjuvant chemotherapy	Yes		18	187	0.416
	No		44	360	

MUC1 mucin 1, *TMA* tissue-microarray, *Ap* apical, *NST* no special type, *ILC* invasive lobular carcinoma, *IMPC* invasive micropapillary carcinoma, *ICC* invasive cribriform carcinoma, *L.I.* labelling index, *ER* estrogen receptor, PgR progesterone receptor
